# Evidence-based nursing practice for the removal of peripheral arterial catheters in adult patients

**DOI:** 10.1186/s12912-026-04999-w

**Published:** 2026-07-06

**Authors:** Yuzhen Ma, Lihuan He, Xin Liu, Luxin Qiu, Jianhong Wan

**Affiliations:** 1 Orthopaedic Trauma Surgery, The Second Qilu Hospital of Shandong University, Jinan, China; 2 Cardiovascular Surgery, The Second Qilu Hospital of Shandong University, 247 Beiyuan Ave, Jinan, Shandong 250033 China

**Keywords:** Evidence translation, Peripheral arterial catheter, Removal, Evidence-based nursing

## Abstract

**Objective:**

To establish a standard procedure for the removal of peripheral arterial catheters (AC) in critically ill adult patients through the application of the best evidence for the removal of AC.

**Methods:**

16 best evidences were summarized through systematic retrieval and quality evaluation of the topics related to the removal of AC. 9 evidence-based quality examination standards were formulated based on clinical scenarios and professional judgment. The review indicators were implemented using the Johns Hopkins nursing evidence-based practice model in the Cardiovascular Surgery Intensive Care Unit.

**Results:**

After the second round of examination, the compliance rate of review indicators was significantly higher than before (*P < 0.05*), and the incidence of complications in patients significantly decreased (*P < 0.05*).

**Conclusion:**

The implementation of the evidence-based practice protocol for the removal of AC in adults has standardized the procedure for AC removal and enhanced the safety of the process.

**Clinical trial number:**

Not applicable.

## Introduction

Peripheral arterial catheters (ACs) are catheters inserted into the arterial lumen via percutaneous arterial puncture. They are used for invasive blood pressure monitoring and facilitate the collection of arterial blood gas samples [1, 2]. ACs are widely used in cardiac surgery intensive care units (CSICUs) to provide accurate and continuous blood pressure monitoring for patients after cardiac surgery [3]. Although arterial catheter (AC) insertion techniques have become increasingly mature [4], the nursing management of ACs remains insufficient compared with that of central venous catheters (CVCs) [5], particularly with regard to catheter removal. In clinical practice, considerably less attention has been paid to AC removal than to AC insertion. In fact, AC removal is not always a risk-free procedure. Improper removal may lead to local ecchymosis, swelling, hematoma, bleeding, thrombosis, distal limb ischemia, pseudoaneurysm, and infection [1, 6]. The severity of these complications can range from mild swelling to conditions requiring surgical intervention [7]. A systematic review by Wang et al. [5] reported that approximately 8 million ACs are used annually in the United States, while nearly 2.5 million are used in Europe [8]. In addition, the incidence of local hematoma after AC removal has been reported to be 1.2% [9]. Local ischemia has also been observed in 0.2% of patients during catheter removal, characterized by symptoms such as pain, localized discoloration, abnormal sensation, swelling, and decreased temperature at and distal to the catheter insertion site [10, 11]. Although the incidence of these complications appears relatively low, the large volume of AC use means that even low-probability events may affect a substantial number of patients.

Existing vascular access management guidelines [12] mainly provide detailed recommendations on the insertion, use, maintenance, and removal of venous catheters, whereas AC removal is only briefly mentioned, without providing evidence-based guidance for the removal procedure. As a result, AC removal practices vary considerably across healthcare institutions and are primarily guided by nursing experience rather than evidence-based recommendations. This gap between practice and evidence is particularly prominent in CSICUs, because patients after cardiac surgery often require anticoagulant therapy, which may increase the risk of bleeding during AC removal; however, this issue has not been adequately addressed in existing guidelines.

There are substantial differences between the removal procedures for venous catheters and ACs. AC removal itself is an independent clinical event that requires specific assessment capabilities and technical skills, including appropriate compression techniques, sufficient compression duration, post-removal peripheral circulation assessment, and individualized risk-control measures. At present, standardized operational protocols for these procedures remain lacking. To address this gap and improve patient experience, we proposed an evidence translation study. This study systematically applied the Johns Hopkins Nursing Evidence-Based Practice Model (JHNEBP) [12] to translate the best available evidence into a standardized AC removal protocol. Unlike previous quality improvement projects conducted in single clinical settings, this study aimed to develop, implement, and evaluate a protocol designed to improve the safety of AC removal in CSICU patients, with explicit assessment of its implementation outcomes, thereby laying the foundation for generating more generalizable knowledge in the future.

## Methods

## Trial design

The current study was a prospective quasi-experimental before-and-after study. This study was conducted from May to December 2024 in the CSICU of a large general hospital in Shandong Province, China. JHNEBP was chosen to guide the evidence-based practice intervention for AC removal. The entire study process strictly followed the PET framework of JHNEBP. The implementation process was carried out and presented in the manuscript according to the following three steps:

### Practice question development

#### Establishment of a multidisciplinary evidence translation project team

All experimental procedures were approved by the Research Ethics Committee of the Second Qilu Hospital of Shandong University (License No. KYLL2024LW038). In accordance with the JHNEBP, a multidisciplinary team was established and a team leader was appointed prior to formulating the research question. The project was led by the head nurse of the CSICU, who served as both the project convener and team leader. A multidisciplinary project team was established in accordance with the stakeholder principle. The head nurse was responsible for organizing project progress meetings, developing action strategies, and monitoring project progress and the implementation of related measures.

The research project team consisted of seven members. One deputy head nurse and one postgraduate student were responsible for conducting evidence searches based on the clinical problem, and independently performed literature appraisal, evidence evaluation, and evidence synthesis. One CSICU intensivist and two senior nurses served as the main implementation personnel, responsible for clinical translation of the evidence as well as data collection and entry.

In cardiac surgery, ACs are commonly inserted by anesthesiologists before surgery. Therefore, one associate chief anesthesiologist was included as a stakeholder. This member participated in project team meetings and was responsible for providing recommendations based on the strength of the evidence, as well as assisting with investigations and opinion collection related to evidence translation. The multidisciplinary team proceeded with the project in a structured manner according to the predetermined schedule and organized the research into a hierarchy using JHNEBP tools.

#### Based on the practice question, the question was structured using the PICOT framework

The inclusion criteria were as follows: (1) Population (P): adult patients (≥ 18 years) with indwelling peripheral arterial catheters; (2) Intervention (I): management related to peripheral arterial catheter removal and replacement; (3) Control (C): none; (4) Outcomes (O): primarily patient-level outcomes, including complications related to peripheral arterial catheter removal, such as bleeding, infection, hematoma, embolism, and the incidence of safety-related adverse events; and (5) Types of evidence (T): secondary literature, including clinical practice guidelines, clinical decision support tools, evidence summaries, systematic reviews, expert consensus statements, and meta-analyses.

The exclusion criteria were as follows: (1) publications in languages other than Chinese or English; (2) duplicate publications or studies with updated versions available; (3) literature types including primary research studies, study protocols, commentaries, and translated articles; and (4) publications with incomplete information or unavailable full texts.

#### Evidence retrieval

**Fig. 1:**
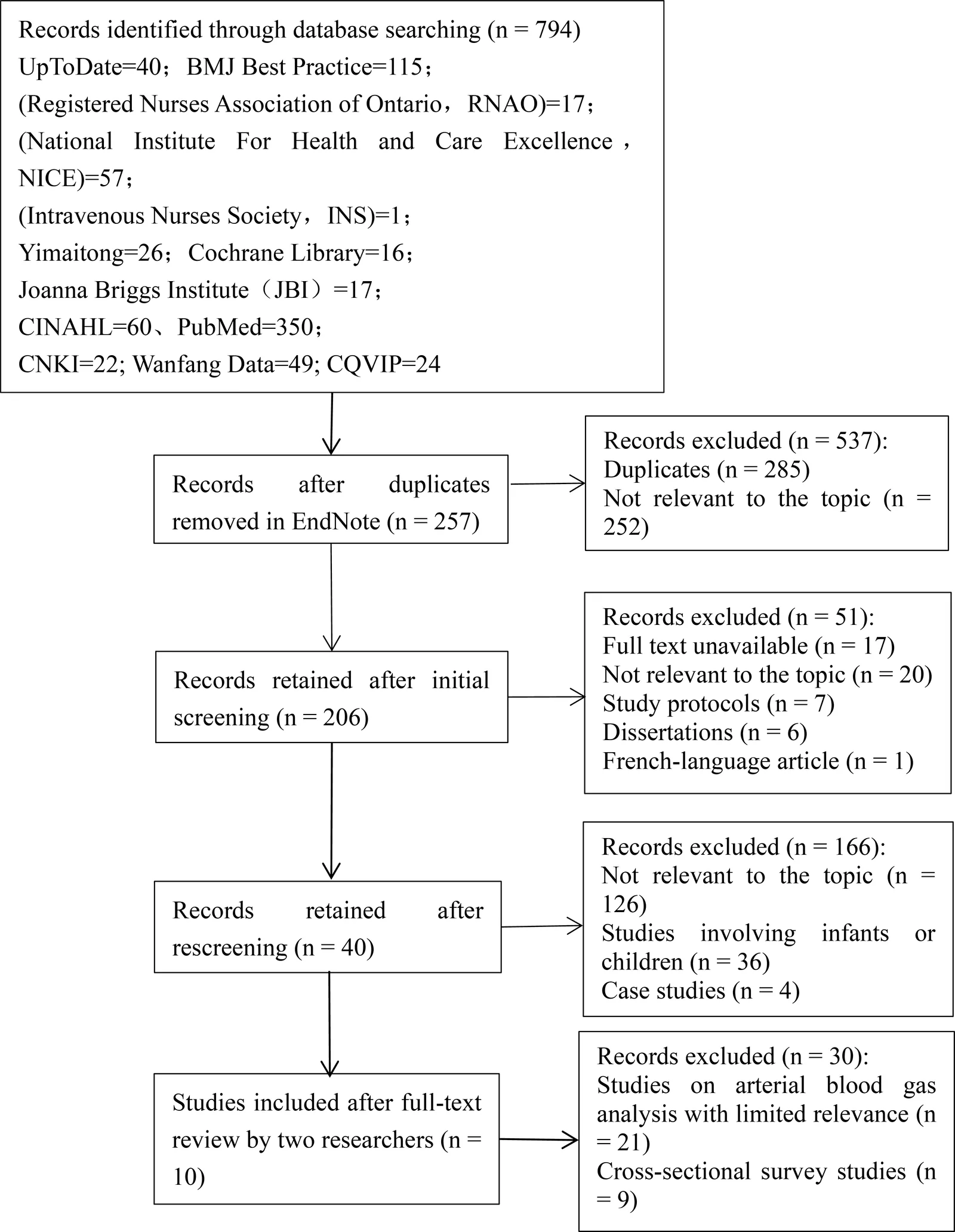
Flow diagram of the literature search and study selection process

Evidence retrieval was conducted in a stepwise manner in accordance with the 6 S evidence model. Notably, a previous systematic review addressing a largely similar PICO question has been published [5], with a literature search period ranging from January 1, 2000 to August 30, 2021. In the present study, the search strategy was updated by extending both the search timeframe and search scope on the basis of the previous review. A comprehensive computerized literature search was performed across authoritative evidence-based resources and bibliographic databases, including UpToDate, BMJ Best Practice, the Registered Nurses’ Association of Ontario (RNAO), the National Institute for Health and Care Excellence (NICE), the Infusion Nurses Society (INS), Yimaitong, the Cochrane Library, the Joanna Briggs Institute (JBI) Evidence-Based Health Care Database, CINAHL, PubMed, China National Knowledge Infrastructure (CNKI), Wanfang Data, and the VIP Database. All databases were searched from inception to August 30, 2023. Database-specific search strategies were developed by combining Medical Subject Headings (MeSH) with free-text terms. The English search terms used in this study were as follows: “arterial catheter*/ arterial line/ A-line/ catheter, indwelling/ arterial cannulation/ vascular access devices/ blood gas analysis”AND“removal”AND“meta analysis/ guideline*/ Evidence summary/ systematic review/ system overview/ clinical practice/ guidelines/ Recommended practice”. The Chinese search terms were as follows: “动脉导管” OR “动脉置管” OR “动脉测压管” OR “血管通路” OR “血管置管” OR “血气分析”) AND (“拔除” OR “移除” OR “拔管” OR “拔出”) AND (“出血” OR “感染” OR “栓塞” OR “风险” OR “堵塞” OR “并发症” OR “安全” OR “意外” OR “不良事件”) AND (“Meta分析” OR “指南” OR “证据总结” OR “系统评价” OR “系统综述” OR “专家共识” OR “临床决策”). The literature screening process is presented in Fig. 1. A total of 794 relevant records were retrieved through the systematic search. Following duplicate removal, preliminary screening, and full-text review, 10 studies were finally included in the evidence synthesis. The retrieved literature was evaluated in two stages. First, methodological quality appraisal was conducted to screen eligible studies. Second, evidence grading was performed to determine the strength of evidence provided by the included studies.

First, in Stage 1, methodological quality appraisal was conducted. According to the type of literature, corresponding quality appraisal tools were used. Guidelines were appraised using the Appraisal of Guidelines for Research and Evaluation II (AGREE II, 2012 version) [13]. Evidence summaries were evaluated using the Critical Appraisal for Summaries of Evidence (CASE) checklist [14]. Systematic reviews were assessed using A MeaSurement Tool to Assess Systematic Reviews 2 (AMSTAR 2) [15]. Expert consensuses were appraised using the Joanna Briggs Institute (JBI) Critical Appraisal Tool for Expert Consensus, 2015 version [16]. For clinical decision-making resources, the original evidence sources were traced, and appropriate appraisal tools were selected according to the type of the original literature.

The methodological quality appraisal was independently performed by two researchers. Any disagreements were resolved through discussion or adjudication by a third party. Studies rated as “eligible” in the methodological quality appraisal were included in the final evidence synthesis.

Second, in Stage 2, evidence grading was performed. For eligible studies that passed the methodological quality appraisal, two researchers independently graded the evidence using the evidence-level tool from the fourth edition of the Johns Hopkins Nursing Evidence-Based Practice Model (JHNEBP) [12], and the level of each item of evidence was determined from Level I to Level V.

After this two-stage screening process, 10 eligible studies were finally included, comprising one clinical decision-making resource [17], three guidelines [11, 18, 19], four evidence summaries [20–23], and two expert consensuses [24, 25]. The results are shown in Table 1.

**Table 1:** Characteristics and quality appraisal of the included studies

No.	Included literature	Publication year	Source	Document type	Evidence level	Quality Rating	Research topic
1	Arthur CT et al. [17]	2022	UpToDate	Clinical decision support	Level IV	A	Arterial Catheterization for Invasive Blood Pressure Monitoring: Removal
2	Gorski LAet al. [11]	2021	INS	Clinical practice guideline	Level IV	A	Removal of Vascular Access Devices
3	O’Grady NPet al. [18]	2011	Yimaitong	Clinical practice guideline	Level IV	B	Practice recommendations for vascular catheters
4	Loveday HPet al. [19]	2014	NICE	Clinical practice guideline	Level IV	A	Catheter replacement strategies
5	Munn Z.et al. [20]	2022	JBI	Evidence summary	Level V	A	Removal of peripheral arterial catheters
6	Pamaiahgari P B. [21]	2023	JBI	Evidence summary	Level V	A	Replacement of peripheral arterial catheters
7	Wang Y et al. [22]	2020	Wanfang Data	Evidence summary	Level V	B	Timing of peripheral arterial catheter removal
8	Guo HH et al. [23]	2021	Wanfang Data	Evidence summary	Level V	B	Timing of arterial pressure monitoring catheter removal
9	Jean-François Timsit, [24]	2020	PUBMED	Expert consensus	Level IV	A	Assessment for arterial catheter removal
10	Ma H et al. [25]	2017	Chinese Journal of Anesthesiology	Expert consensus	Level IV	B	Complications and management of radial artery catheterization for pressure monitoring

### Evidence appraisal and evidence synthesis

Based on the evidence level and methodological quality grading of the included literature, all appraised evidence items were summarized, and the content, source, and level of each item of evidence were clearly identified. For conflicting evidence, the principle of level priority was applied. Specifically, when higher-level evidence conflicted with lower-level evidence, the conclusions of the higher-level evidence were adopted in principle, unless the higher-level evidence had a serious risk of bias.

For conflicts between evidence items at the same level, priority was given to studies with a more recent publication year, larger sample size, and higher methodological quality score. Any remaining ambiguities were resolved through collective discussion and consensus within the multidisciplinary project team.

Ultimately, 16 evidence items across five themes were included. The results are shown in Table 2.

**Table 2:** Evidence-based audit criteria and methods for peripheral arterial catheter removal in adults

Evidence domain	NO.	Evidence statement	Evidence level	Quality Rating
Assessment of the timing of peripheral arterial catheter removal	1	The necessity of maintaining an arterial catheter should be assessed on a daily basis, with a thorough evaluation of the potential benefits and risks. When continued catheterization is no longer indicated, the arterial catheter should be removed as early as possible [11, 17, 20–24].	Level IV	A
	2	An arterial catheter should be removed immediately in the presence of clear signs of infection, catheter dysfunction, the occurrence of complications (including occlusion, hematoma, or circulatory impairment), or when the arterial catheter is no longer clinically indicated [11,19,21,23–25].	Level IV	A
	3	If an arterial catheter is inserted under emergency conditions without strict adherence to aseptic technique, the indwelling duration should not exceed 48 h [18–20].	Level IV	A
Preparation prior to peripheral arterial catheter removal	4	Prior to arterial catheter removal, the international normalized ratio (INR), activated partial thromboplastin time (aPTT), and platelet count should be assessed, and the use of anticoagulant or antiplatelet agents should be confirmed [17,24].	Level IV	A
	5	Before performing arterial catheter removal, hand hygiene should be completed, followed by donning sterile gloves and wearing a sterile isolation gown [17].	Level IV	A
	6	Prior to arterial catheter removal, the catheter insertion site should be disinfected with chlorhexidine [11,17,22].	Level IV	A
	7	Prior to arterial catheter removal, the arterial catheter should be flushed to minimize the risk of blood splashing during removal [17].	Level IV	A
Peripheral arterial catheter removal procedure	8	A sterile dressing should be applied over the arterial puncture site with manual compression, followed by slow and gentle withdrawal of the catheter [11,14].	Level IV	A
	9	During catheter removal, both the arterial puncture site and the skin entry site should be compressed simultaneously, and hemostasis should be achieved through manual pressure [11,14].	Level IV	A
Duration of post-removal compression	10	After removal of a radial arterial catheter, manual compression should generally be maintained for 5 min, whereas compression should be continued for 10 min following femoral arterial catheter removal [17].	Level IV	B
	11	If any abnormalities are present in the international normalized ratio (INR), activated partial thromboplastin time (aPTT), or platelet count, or if the patient is receiving antiplatelet therapy, the duration of compression should be prolonged. Following radial arterial catheter removal, manual compression should be maintained for 10 min, whereas 20 min of compression is recommended after femoral arterial catheter removal, or alternatively, a mechanical compression device may be used for femoral artery hemostasis [17,20].	Level IV	A
	12	If oozing persists after the recommended compression duration, manual compression should be continued for an additional 5 min, followed by reassessment [17].	Level IV	A
Key considerations after peripheral arterial catheter removal	13	After catheter removal, the catheter should be inspected to ensure its integrity. If catheter fracture is identified, compression should be applied distal to the catheter insertion site. Catheter fragment embolization may obstruct distal limb circulation and requires prompt referral to surgical services [11,17,19].	Level IV	A
	14	Patients who have undergone femoral arterial catheterization should avoid hip flexion for at least 2 h following catheter removal [18,25].	Level IV	A
	15	Patients should be instructed to avoid forceful gripping with the limb on the puncture side and to refrain from noninvasive blood pressure measurement on that limb within 2 h after catheter removal. The puncture site should be closely monitored for signs of bleeding or subcutaneous hematoma formation [17,25].	Level IV	A
	16	Fifteen minutes after catheter removal, the puncture site and distal pulses should be re-examined and documented to assess for the presence of hematoma or signs of limb ischemia. If abnormalities in circulation and/or sensation are detected, the physician should be promptly notified [11,17,19].	Level IV	A

### Evidence translation into practice

#### Baseline audit

To understand the current status of clinical practice before evidence translation and identify weak links in evidence implementation, the CSICU of a Grade-A tertiary hospital in Shandong Province was selected as the evidence translation site. Support was obtained from the departmental medical director and the director of the nursing department.

Based on the evidence synthesis, the project team developed an audit checklist for the removal of peripheral ACs in adult patients. Five external experts, including one deputy director of the nursing department, two experts in evidence-based nursing methodology, and two cardiovascular surgeons, were invited to evaluate the content validity of the audit indicators. A four-point rating scale was used, with 1 indicating “not relevant” and 4 indicating “highly relevant”. The item-level content validity index (I-CVI) and scale-level content validity index (S-CVI) were calculated. The predefined criteria were I-CVI ≥ 0.78 and S-CVI ≥ 0.90. The results showed that the I-CVI values of the nine indicators ranged from 0.80 to 1.00, and the S-CVI was 0.93.

The correspondence between the audit indicators and the evidence items is shown in Table 3. The audit methods included on-site observation, review of nursing records, interviews with medical staff, and interviews with patients. The principles for method selection were as follows: on-site observation was used for process indicators; nursing records were reviewed for outcome indicators; and interviews were conducted for outcome indicators that did not require documentation. These three methods complemented one another.

A prospective study was conducted to identify gaps between current practice and the best available evidence. The observers were a CSICU intensivist and a postgraduate nursing student from the project team. This arrangement ensured that the observation time was synchronized with the operators and that the observers had no direct conflict of interest with the operators, thereby minimizing the Hawthorne effect and potential conflicts of interest as much as possible.

From May to July 2024, the observers conducted a baseline clinical audit among adult patients admitted to the CSICU who underwent peripheral AC removal. They also observed and recorded the occurrence of complications after catheter removal. The specific audit indicators and methods are presented in Table 3. The audit showed substantial gaps between current practice and Evidence items 3, 4, 5, 6, 7, 10, 11, 14, 15, and 16. The detailed data are shown in Table 5.

**Table 3:** Audit criteria and methods for peripheral arterial catheter removal in adult patients (final version)

Evidence domain	NO.	Evidence statement	Audit criteria	Audit criterion	Calculation formula	Audit methods
Assessment of the timing of peripheral arterial catheter removal	1	The necessity of maintaining an arterial catheter should be assessed on a daily basis, with a thorough evaluation of the potential benefits and risks. When continued catheterization is no longer indicated, the arterial catheter should be removed as early as possible [11, 17, 20–24].	The responsible nurse assesses the necessity of catheter indwelling and catheter function within 1 h after shift handover each day, and removes the catheter promptly if complications occur.	Daily assessment rate of catheter necessity	Daily assessment rate = (Number of patients assessed daily for catheter removal indications / Number of patients with indwelling catheters) × 100%	On-site investigation, nursing record review, and nurse interviews
	2	An arterial catheter should be removed immediately in the presence of clear signs of infection, catheter dysfunction, the occurrence of complications (including occlusion, hematoma, or circulatory impairment), or when the arterial catheter is no longer clinically indicated [11,19,21,23–25].				
	3	If an arterial catheter is inserted under emergency conditions without strict adherence to aseptic technique, the indwelling duration should not exceed 48 h [18–20].	Medical staff assess whether aseptic technique was followed during arterial catheter insertion; if not, the catheter is removed or replaced within 48 h.	Daily assessment rate of catheter necessity	Daily assessment rate = (Number of patients assessed daily for catheter removal or replacement indications / Number of patients whose catheter insertion did not comply with maximal sterile barrier precautions) × 100%	On-site observation and interviews
Preparation prior to peripheral arterial catheter removal	4	Prior to arterial catheter removal, the international normalized ratio (INR), activated partial thromboplastin time (aPTT), and platelet count should be assessed, and the use of anticoagulant or antiplatelet agents should be confirmed [17,24].	Before arterial catheter removal, it should be confirmed whether the patient is receiving anticoagulant or antiplatelet medications [17,24]. If the patient is not receiving such medications, the most recent coagulation test results should be reviewed for abnormalities.	Pre-removal confirmation rate of anticoagulant use and coagulation parameters	Confirmation rate = (Number of catheter removal cases in which anticoagulant medication use and pre-removal coagulation indicators were confirmed / Total number of catheter removal cases) × 100%.	On-site observation and interviews
	5	Before performing arterial catheter removal, hand hygiene should be completed, followed by donning sterile gloves. [17].	Before catheter removal, hand hygiene is performed and gloves. The dressing is removed using a 0°–180° technique, after which the insertion site is disinfected with chlorhexidine. The arterial catheter is flushed, and a sterile dressing measuring at least 4 × 4 cm is placed over the skin puncture site and arterial puncture site for compression while the catheter is slowly removed.	Standardized pre-removal preparation is performed before peripheral arterial catheter removal.	Completion of key procedural steps during catheter removal was assessed, and any omitted step was recorded. The key steps were as follows: ① hand hygiene, gloves; ② dressing removal in a 0°–180° direction; ③ chlorhexidine disinfection of the puncture site; ④ arterial catheter flushing; ⑤ application of a sterile dressing ≥ 4 × 4 cm to compress the puncture site; and ⑥ slow catheter removal.	On-site observation and interviews
	6	Prior to arterial catheter removal, the catheter insertion site should be disinfected with chlorhexidine [11,17,22].				
	7	Prior to arterial catheter removal, the arterial catheter should be flushed to minimize the risk of blood splashing during removal [17].				
Peripheral arterial catheter removal procedure	8	A sterile dressing should be applied over the arterial puncture site with manual compression, followed by slow and gentle withdrawal of the catheter[11,14].				
	9	During catheter removal, both the arterial puncture site and the skin entry site should be compressed simultaneously, and hemostasis should be achieved through manual pressure [11,14].				
Duration of post-removal compression	10	After removal of a radial arterial catheter, manual compression should generally be maintained for 5 min, whereas compression should be continued for 10 min following femoral arterial catheter removal [17].	The compression duration after catheter removal is in accordance with the specified requirements.	Compliance rate for post-removal compression duration	Compliance rate = (Number of catheter removal cases with adequate compression duration / Total number of catheter removal cases) × 100%	On-site observation and interviews
	11	If any abnormalities are present in the international normalized ratio (INR), activated partial thromboplastin time (aPTT), or platelet count, or if the patient is receiving antiplatelet therapy, the duration of compression should be prolonged. Following radial arterial catheter removal, manual compression should be maintained for 10 min, whereas 20 min of compression is recommended after femoral arterial catheter removal, or alternatively, a mechanical compression device may be used for femoral artery hemostasis [17,20].	Before arterial catheter removal, it should be confirmed whether the patient is receiving anticoagulant or antiplatelet medications [17,24]. If the patient is not receiving such medications, the most recent coagulation test results should be reviewed for abnormalities.	Pre-removal confirmation rate of anticoagulant use and coagulation parameters.	Confirmation rate = (Number of catheter removal cases in which anticoagulant medication use and pre-removal coagulation indicators were confirmed / Total number of catheter removal cases) × 100%.	On-site observation and interviews
			The compression duration after catheter removal is in accordance with the specified requirements.	Compliance rate for post-removal compression duration	(Number of catheter removal cases with adequate compression duration / Total number of catheter removal cases) × 100%	
	12	If oozing persists after the recommended compression duration, manual compression should be continued for an additional 5 min, followed by reassessment [17].	The compression duration after catheter removal is in accordance with the specified requirements.	Compliance rate for post-removal compression duration	Compliance rate for post-removal compression duration = (Number of catheter removal cases with adequate compression duration / Total number of catheter removal cases) × 100%	On-site observation and interviews
Key considerations after peripheral arterial catheter removal	13	After catheter removal, the catheter should be inspected to ensure its integrity. If catheter fracture is identified, compression should be applied distal to the catheter insertion site. Catheter fragment embolization may obstruct distal limb circulation and requires prompt referral to surgical services [11,17,19].	Medical staff check catheter integrity after removal and apply compression to the distal side of the catheter site if breakage occurs.	Catheter integrity check implementation rate and nurses’ knowledge awareness rate	(Number of cases in which catheter integrity was checked after catheter removal / Total number of catheter removal cases) × 100%; nurses were interviewed regarding their knowledge of catheter breakage.	On-site observation and interviews
	14	Patients who have undergone femoral arterial catheterization should avoid hip flexion for at least 2 h following catheter removal [18,25].	The patient understands the requirements for post-removal positioning and management of the puncture-side limb.		Patient interviews regarding their knowledge of post-removal care specified in Evidence item 14.	On-site observation and interviews
	15	Patients should be instructed to avoid forceful gripping with the limb on the puncture side and to refrain from noninvasive blood pressure measurement on that limb within 2 h after catheter removal. The puncture site should be closely monitored for signs of bleeding or subcutaneous hematoma formation [17,25].		Patient awareness rate of post-removal care knowledge	Patient interviews regarding their knowledge of post-removal care specified in Evidence item 15.	On-site observation and interviews
	16	Fifteen minutes after catheter removal, the puncture site and distal pulses should be re-examined and documented to assess for the presence of hematoma or signs of limb ischemia. If abnormalities in circulation and/or sensation are detected, the physician should be promptly notified [11,17,19].	Medical staff assess the puncture site and distal pulse following catheter removal.	Post-removal nursing assessment implementation rate	(Number of cases in which the puncture site and distal pulse were assessed after catheter removal / Total number of catheter removal cases) × 100%	On-site observation and interviews
			The condition of the puncture site and distal pulse assessed after catheter removal is documented in the nursing record.	Post-removal nursing record completeness rate	(Number of cases with complete post-removal records / Total number of catheter removal cases) × 100%	

#### Pilot implementation

After obtaining the baseline data, the project team held a meeting to report the findings and develop the next-stage work plan, with the main focus on formulating the training program. The training program included the development of operating procedures and standards by project team members. Theoretical training and procedural demonstrations for medical staff in the department were completed within one week. In addition to in-department training, the operating procedures and standards were reviewed and approved by the Technical Operation Group of the Nursing Department, followed by one online training session delivered through the 512 Training software.

After two rounds of departmental training and assessment, a two-week small-sample pilot study was conducted from August 12 to August 25, 2024, involving 22 patient episodes. The main purpose of the pilot study was to identify and address barriers and optimize the implementation strategies. The CSICU head nurse and deputy head nurse served as observers and conducted on-site assessments of the implementation of evidence translation after training. Semi-structured interviews were also conducted with stakeholders, including physicians, nurses, and relevant patients, to identify barriers to evidence translation and further optimize the implementation strategies.

Evidence items 7 and 10 showed high acceptability after training. Therefore, expert consultation was conducted for the remaining eight evidence items that showed relatively large gaps between evidence and clinical practice. In addition to the project team members, the head of the hospital’s Vascular Access Nursing Academic Group and the ICU head nurse were invited to participate.

Based on the JHNEBP model, and with reference to the FAME framework—which evaluates evidence in terms of feasibility, appropriateness, meaningfulness, and effectiveness—we assessed the applicability of the synthesized evidence to the local clinical setting, Meanwhile, benefits and risks, resource readiness [26], compatibility with organizational culture, feasibility of available resources, and stakeholder willingness were also considered. Finally, the evidence implementation strategies were iteratively optimized. In particular, for the initial Evidence item 5, sterile gloves and a sterile isolation gown were recommended after hand hygiene before AC removal. Although this recommendation was indicated in the clinical decision support resource, operator acceptance was low, and staff expressed concerns regarding the additional expenditure on disposable consumables and the necessity of this requirement. The original evidence item used the wording “recommended,” which allowed considerable discretion in actual clinical practice and resulted in relatively low compliance. After expert consultation, this evidence item was revised as follows: sterile gloves should be worn after hand hygiene before AC removal, while the requirement for wearing a sterile isolation gown was removed. In addition, nurses had misconceptions regarding the initial Evidence items 4 and 11. Some nurses believed that because AC removal was necessary regardless of whether patients were receiving anticoagulants or had abnormal coagulation results, it would be sufficient to simply prolong the compression duration without reviewing the patients’ coagulation results. However, unnecessary prolonged compression is time-consuming; hand and arm fatigue, together with inconsistent pressure, may lead to hematoma and/or thrombosis [27]; and prolonged compression may also cause temporary arterial occlusion [28]. Given that radial artery occlusion caused by compression has been reported, the use of a “non-occlusive hemostasis” technique during compression is recommended, which refers to applying the minimum pressure required to prevent arterial bleeding while maintaining blood flow through the radial artery [29]. Through data analysis, the project team found that 77.78% of patients were receiving anticoagulant or antiplatelet medications at the time of catheter removal, and medication orders were more readily accessible to nurses. Therefore, a medication-first strategy was adopted. If a patient was receiving antiplatelet medication, nurses could directly prolong the duration of “non-occlusive hemostasis” compression without further checking the international normalized ratio, activated partial thromboplastin time, or platelet count. The details are shown in Table 4.

After iterative optimization of the evidence implementation strategies was completed, an expanded project team meeting was organized. In addition to the project team members, the CSICU medical team and nursing team leaders also participated. Evidence items with poor implementation were reviewed again according to the solutions proposed through expert consultation and the revised procedures. Training on the iteratively optimized strategies was then conducted again according to the contents presented in Table 4.

**Table 4:** Framework for addressing evidence items with poor implementation

evidence items with suboptimal implementation	Barrier type	Implementation strategy	Revisions	Description of Revisions
Evidence 3: Timing of catheter removal for emergency catheterization patients	Insufficient staff awareness of risks associated with emergency catheterization	Data-based persuasion: provide AC-related infection rate data; automatic information-system pop-up reminders	Evidence retained; education strengthened	—
Evidence items 4/11: Before AC removal, anticoagulant or antiplatelet medication use should be confirmed first [17,24]. If not used, INR, aPTT, and platelet count should be checked.	Staff time burden: time-consuming retrieval of patients’ laboratory results from the device terminal, leading to low implementation compliance.	Revised to: confirm whether the patient is using anticoagulant or antiplatelet medications before arterial catheter removal. If yes, prolong the compression duration directly; if no, review coagulation test results.	Revision: “anticoagulant medication use first” replaced routine laboratory result retrieval.	After expert consultation, the original evidence item was revised as shown in the first column. This revision ensures prolonged compression for patients at high risk of bleeding, while reducing unnecessary time spent retrieving coagulation test results and improving clinical feasibility.
Evidence item 5: Before AC removal, sterile gloves are recommended.	Increased expenditure on disposable consumables, as sterile gloves are more costly than clean gloves; concerns regarding the necessity of this requirement; and considerable operator discretion due to the wording “are recommended,” resulting in low compliance.	Revised to: sterile gloves should be worn after hand hygiene before AC removal.	Modified recommendation	After expert consultation, the wording was strengthened to “sterile gloves should be worn” to reduce ambiguity. When strict hand hygiene is performed and sterile gloves are worn, omitting the sterile isolation gown was considered unlikely to increase infection risk. Removing this requirement may improve compliance; therefore, the recommendation to wear a sterile isolation gown was removed.
Evidence item 6: Disinfection of the catheter puncture site with chlorhexidine before catheter removal	Process barrier: limited convenience and accessibility for nurses independently using sterile cotton swabs dipped in chlorhexidine disinfectant.	Modified to the use of a central line maintenance kit with chlorhexidine gluconate (CHG kit).	Evidence retained	—
Evidence item 10: Compliance with the required compression duration	Process barrier: nurses reported that extending compression duration interfered with the timely completion of other nursing tasks.	Nurse-led selection of catheter removal timing; initiation of a collaboration mechanism during local compression to ensure uninterrupted compression.	Evidence retained	—
Evidence items 14 and 15: Patient awareness of self-care points after peripheral arterial catheter removal	Low risk perception among patients	Early-stage patient education: include AC indwelling and removal in preoperative education, and provide written instructions on precautions for radial and femoral arterial catheterization and removal.	The evidence item was retained, and patient education was provided earlier in the care process.	—
Evidence item 16: Reassessment and documentation of the puncture site and distal pulse 15 min after catheter removal	Low risk perception among staff	Case-based education using previous cases; automated data collection embedded in the information system	The evidence item was retained, and education was strengthened.	—

#### Effect evaluation phase

A pre–post controlled quasi-experimental study was conducted to evaluate the effects of the evidence translation program on process and outcome indicators. The same two observers from the project team, namely a CSICU intensivist and a postgraduate nursing student, audited peripheral AC removal procedures performed in CSICU patients from October to December 2024. These data were compared with those collected from May to July 2024.

The evaluation indicators included the following:

① Evidence implementation: implementation of each evidence item was recorded as “Y”, whereas non-implementation or partial implementation was recorded as “N”. The evidence implementation rate was calculated as follows: evidence implementation rate = number of implemented cases / total number of catheter removal cases × 100%.

② Patient-related complications: these included puncture-site bleeding/oozing, defined as visible active oozing around the puncture site; puncture-site ecchymosis, defined as visible bruising around the puncture site with a diameter > 2 cm [30]; subcutaneous hematoma, defined as a visible hematoma around the puncture site with a diameter > 2 cm; distal limb ischemia, defined as mottling, ischemia, necrosis, or other ischemic manifestations in the distal limb on the puncture side, after excluding factors such as bilateral distal limb ischemia caused by high-dose vasoactive drug use; and thrombosis, defined as visible thrombus during blood sampling, catheter removal, or at the puncture site [30].

③ Other clinical data: compression duration and number of compression attempts were recorded. Compression duration was prolonged for all patients receiving antiplatelet or anticoagulant medications [17]. When coagulation test results were reviewed, the test closest to the time of catheter removal was used. Any abnormal result in the international normalized ratio, activated partial thromboplastin time, or platelet count was considered abnormal.

### Statistical analysis

Data were analyzed using SPSS version 27.0. Categorical variables are presented as n (%), and between-group comparisons were performed using the chi-square test or Fisher’s exact test, as appropriate. Continuous variables with a normal distribution are presented as mean ± standard deviation, and comparisons between two groups were performed using the t-test. Continuous variables with a non-normal distribution were compared between groups using the rank-sum test. A P value < 0.05 was considered statistically significant.

This study was a pre–post controlled quasi-experimental study, and no causal hypothesis was prespecified. Given the inherent risk of bias associated with the pre–post controlled design, including temporal effects and the Hawthorne effect, this study aimed to provide practical data to inform more rigorous study designs in the future.

## Results

### General characteristics of the study population

#### Comparison of general characteristics between the two groups

A total of 143 patients were included in this study, including 69 patients in the control group and 74 patients in the intervention group. There were no statistically significant differences in baseline characteristics between the two groups, as shown in Table 5.

**Table 5:** Comparison of baseline characteristics between the control and intervention groups

Variable			Total (*n* = 143)	Control group (*n* = 69)	Intervention group (*n* = 74)	Test statistic (χ² / t)	*P* value
Sex	Male		105	51(73.9%)	54(73.0%)	0^a^	1
	Female		38	18(26.1%)	20(27.0%)		
Age (years)			59.6	58.9 ± 16.0	60.4 ± 11.8	-0.65^b^	0.516
Primary diagnosis	Coronary artery disease		57	28(40.57%)	29(39.19%)	2.54^a^	0.111
	Valvular heart disease		26	14(20.29%)	12(16.22%)		
	Aortic dissection		48	19(27.54%)	29(39.19%)		
	Adult congenital heart disease		8	5(7.25%)	3(4.05%)		
	Other		4	3(4.35%)	1(1.35%)		
Arterial catheter insertion site	Left radial artery		105	50(72.5%)	55(74.3%)	0.06^a^	0.969
	Right radial artery		36	18(26.1%)	18(24.3%)		
	Dorsalis pedis artery		2	1(1.4%)	1(1.4%)		
Catheter dwell time (h)				105.6 ± 73.0	99.8 ± 81.9	0.45^b^	0.655
Coagulation time		Normal	26	13(18.8%)	13(17.6%)	0^a^	1
		Abnormal	117	56(81.2%)	61(82.4%)		
Use of antiplatelet agents		Yes	112	55(79.7%)	57(77.03%)	0.03^a^	0.852
		No	31	14(20.3%)	17(22.97%)		

Note: a indicates the chi-square test, and b indicates the *t* test

#### Implementation rates of audit indicators, specific compression duration and number of compression attempts, and incidence of patient-related complications before and after evidence translation

Compared with the pre-translation period, the implementation rates of audit indicators increased significantly after evidence translation (Table 6). The duration of local compression after catheter removal was significantly longer in the intervention group than in the control group, while no significant difference was observed in the number of compression attempts (Table 7). Notably, the overall incidence of complications at the peripheral AC insertion site and the incidence of local bleeding/oozing were significantly reduced (Table 8).

**Table 6:** Comparison of implementation rates of process audit indicators before and after evidence translation

Process indicator	Pre-translation period		Post-translation period		χ² value	*P* value
	Implemented (*n*, %)	Not implemented (*n*, %)	Implemented (*n*, %)	Not implemented (*n*, %)		
Audit criterion 1	61(88.4%)	8(11.6%)	74(100%)	0(0%)	7.03	0.008
Audit criterion 2	47(68.1%)	22(31.9%)	72(97.3%)	2(2.7%)	19.73	<0.001
Audit criterion 3	33(47.8%)	36(52.2%)	70(94.6%)	4(5.4%)	36.48	<0.001
Audit criterion 4	46(66.7%)	23(33.3%)	70(94.6%)	4(5.4%)	16.41	<0.001
Audit criterion 5	23(33.3%)	46(66.7%)	67(90.5%)	7(9.5%)	50.42	<0.001
Audit criterion 6	65(94.2%)	4(5.8%)	74(100%)	0(0%)	2.54	0.111
Audit criterion 7	47(68.1%)	22(31.9%)	67(90.5%)	7(9.5%)	9.76	0.002
Audit criterion 8	48(69.6%)	21(30.4%)	74(100%)	0(0%)	24.03	<0.001
Audit criterion 9	0(0%)	69(100%)	69(93.2%)	5(6.8%)	120.62	<0.001

**Table 7:** Comparison of local compression duration and number of compression attempts after catheter removal between the two groups

Variable	Control group (*n* = 69)	Intervention group (*n* = 74)	t value	*P* value
Compression duration (min)	6.9 ± 1.9	9.6 ± 2.8	-8.66	<0.001
Number of compression attempts	1.2 ± 0.5	1.1 ± 0.4	1.23	0.219

**Table 8:** Incidence of patient-related complications in the control and intervention groups

Complication	Total (*n* = 143)	Control group (*n* = 69)	Intervention group (*n* = 74)	χ² value	*P* value
Total number of complications	19(13.29%)	17(24.6%)	2(2.7%)	14.77	<0.001
Puncture site bleeding/oozing	12(8.4%)	11(15.9%)	1(1.4%)		0.008
Puncture site ecchymosis	4(2.8%)	3(4.4%)	1(1.4%)		0.34
Subcutaneous hematoma	2(1.4%)	2(2.9%)	0(0%)		0.44
Thrombosis	1(0.7%)	1(1.4%)	0(0%)		0.47

Note: Except for the chi-square test, Fisher’s exact test was used for all other comparisons

## Discussion

### Analysis of implementation effects before and after the evidence translation project

Invasive catheters have long been a common source of complications in intensive care units and a key focus of nursing management [31]. This study focused on process management for the safe removal of peripheral ACs. Through the implementation of this evidence translation project, nurses’ compliance with standardized peripheral AC removal procedures improved compared with the pre-translation period. As shown in Table 6, the implementation rates of all nine audit indicators increased. In particular, the implementation rate of Indicator 9 increased sharply from 0% to 93.2%, which may be attributed to the integration of evidence translation content into the information system for automated data collection. Meanwhile, the implementation rates of Evidence items 1, 6, and 8 reached 100%.

As shown in Table 8, the incidence of local puncture-site bleeding or oozing, which was the most common complication after AC removal, decreased from 15.9% in the control group to 1.4% in the intervention group. The overall complication rate decreased from 24.6% in the control group to 2.7% in the intervention group. These results suggest that although the mean compression duration increased by 162 s compared with that before the evidence audit, the reduction in bleeding or oozing may also have benefited from the prolonged compression time after catheter removal.

Previous research has indicated that implementation of evidence translation projects can help improve patient catheter safety outcomes and nursing management efficiency, and may reduce catheter-related infection rates [24]. Although this study had certain objective limitations, the project implementation still demonstrated improvements in clinical outcomes. Because this study adopted a pre–post controlled design and lacked a concurrent control group, the observed improvements cannot be directly attributed to the evidence translation project itself. Nevertheless, the improvements in the observed data suggest a beneficial association between structured catheter removal procedures, standardized compression techniques, departmental culture development, and improved safety of catheter removal.

### Departmental culture and system support are critical for sustaining evidence translation projects

The internal facilitating factors emphasized in the JHNEBP model, including departmental culture, team environment, and equipment resources, are essential for the successful implementation of evidence-based practice projects. Culture represents the collective ideals, beliefs, ethical norms, value standards, and behavioral orientations shared by team members. Without a culture that supports compatibility, innovation, and change within the team, it is difficult to promote the orderly and sustainable development of evidence-based practice projects.

When barriers arise during project implementation, it is important to understand the real concerns of practitioners and address them accordingly. For example, issues related to the use of chlorhexidine and compression duration were discussed and optimized, which helped gain broad recognition from stakeholders and facilitated the resolution of barriers. The team should also carefully consider the human, material, and financial resources required for the implementation action plan. In this study, support and assistance were obtained from the medical team, anesthesiology department, nursing department, and pricing office, ensuring the supply of personnel, financial, and material resources, as well as recognition and support for subsequent technical changes.

In particular, regarding nursing documentation, simply requiring nurses to additionally record the puncture site and distal pulse after peripheral AC removal could be misinterpreted by some nurses as increasing non-direct nursing time, thereby reducing compliance. This problem was effectively addressed with the support of the information system.

In addition, the controversy regarding compression duration was resolved through a dialectical approach. For patients receiving antiplatelet or anticoagulant medications, the duration of “non-occlusive hemostasis” compression could be directly prolonged. “Non-occlusive hemostasis” refers to the minimum compression pressure required to prevent arterial bleeding while maintaining blood flow through the radial artery [29]. Relevant training provided nurses with a clear basis for clinical practice.

During the semi-structured interviews, CSICU nurses reported that prolonged compression after catheter removal delayed the completion of other nursing tasks, which reflected a practical concern during project implementation. To address this issue, the team adopted the following measures: nurses were allowed to independently select the timing of catheter removal according to their workload during the shift; and a collaboration mechanism was initiated during local compression to ensure that compression was not interrupted. These measures helped resolve this practical contradiction.

In evidence translation projects, nurses’ active participation and multidisciplinary collaboration are key to ensuring effective implementation and long-term sustainability [32]. When a routine communication and collaboration mechanism is established around a specific clinical issue, it not only facilitates the standardization of procedural workflows but also enhances the team’s understanding of shared goals and collective commitment, thereby promoting the development of a culture of continuous improvement [33].

### Future research directions

This study adopted a single-center, non-randomized pre–post controlled design. In the future, multicenter randomized controlled trials may be conducted to explore the effects of different compression strategies, such as compression assisted by pressure devices. In addition, further studies may investigate the interactions among different anticoagulant/antiplatelet regimens, the degree of coagulation dysfunction, and optimal compression strategies, thereby facilitating the development of more individualized post-removal action strategies.

From the perspective of implementation science, key factors affecting the long-term sustainability and dissemination of this evidence translation project, such as organizational culture, leadership, and system support, should be systematically evaluated. This may provide a more comprehensive strategic basis for the successful implementation and continuous improvement of similar catheter safety programs in different healthcare settings.

During evidence dissemination, new challenges will continue to emerge, such as the ratio between AC size and patients’ vascular diameter, as well as the appropriate compression pressure after peripheral AC removal [34]. These issues require further in-depth investigation.

## Limitations

### Limitations related to the single-center design and sample size

This study was conducted only in the CSICU of a single Grade-A tertiary hospital, with a relatively limited sample size (*n* = 143), and no a priori sample size calculation was performed. For complications with low incidence, such as pseudoaneurysm and distal ischemia, the current sample size was insufficient to fully evaluate the safety of the program. Therefore, caution should be exercised when generalizing the findings to general ICUs, surgical ICUs, or hospitals of other levels.

### Limitations in causal inference due to the study design

This study adopted a non-randomized pre–post controlled design without a concurrent control group. Therefore, causal inference may be limited because potential confounding factors, such as temporal effects and the Hawthorne effect, could not be completely excluded.

### Lack of cost-effectiveness evaluation

This study did not systematically collect implementation costs related to the evidence translation project, such as training hours and observer time, and no health economic evaluation was conducted.

## Conclusion

This study implemented an evidence translation project on peripheral AC removal based on the fourth edition of the JHNEBP framework [12]. The pre–post evaluation conducted in the CSICU setting showed improved practice compliance, with the implementation rates of all nine audit indicators increasing compared with the baseline audit. Regarding safety outcomes, the overall complication rate decreased from 24.6% in the control group to 2.7% in the intervention group. Because this study adopted a pre–post controlled design and lacked a concurrent control group, these outcomes cannot be directly attributed to the evidence translation project itself. However, the observed trend suggests that structured catheter removal procedures, standardized compression techniques, and the development of a departmental evidence-based culture may be associated with improved safety of catheter removal.

The implementation of this evidence translation project indicates that translating best evidence into clinical practice requires systematic methodological support, such as the JHNEBP framework, multidisciplinary team collaboration, and continuous quality monitoring. It is recommended that this program be incorporated into the standard operating procedures within the CSICU of our institution. Before validation through multicenter prospective studies, the practical components of this program may serve as a reference for other medical centers.

## Data Availability

The datasets generated and/or analyzed during the present study are available from the corresponding author upon reasonable request.

## Abbreviations

AC: Arterial catheters
ACs: Arterial catheters
CSICUs: Cardiac surgery intensive care units
CVCs: Central venous catheters
RNAO: Registered Nurses’ Association of Ontario
INS: Infusion Nurses Society
NICE: National Institute for Health and Care Excellence
JBI: Joanna Briggs Institute
CNKI: China National Knowledge Infrastructure
MeSH: Medical Subject Headings
AGREE II: Appraisal of Guidelines for Research and Evaluation II
CASE: Critical Appraisal for Summaries of Evidence
AMSTAR 2: A MeaSurement Tool to Assess Systematic Reviews 2
INR: International normalized ratio
aPTT: Activated partial thromboplastin time
I-CVI: Item-level content validity index
S-CVI: Scale-level content validity index
